# Cytotoxic Mechanism of Sphaerodactylomelol, an Uncommon Bromoditerpene Isolated from *Sphaerococcus coronopifolius*

**DOI:** 10.3390/molecules26051374

**Published:** 2021-03-04

**Authors:** Celso Alves, Joana Silva, Susete Pinteus, Eva Alonso, Rebeca Alvariño, Adriana Duarte, Diorge Marmitt, Márcia Inês Goettert, Helena Gaspar, Amparo Alfonso, Maria C. Alpoim, Luis M. Botana, Rui Pedrosa

**Affiliations:** 1 MARE—Marine and Environmental Sciences Centre, Politécnico de Leiria, 2520-630 Peniche, Portugal; joana.m.silva@ipleiria.pt (J.S.); susete.pinteus@ipleiria.pt (S.P.); adrianajesusduarte.96@gmail.com (A.D.); 2 Departament of Pharmacology, Faculty of Veterinary, University of Santiago de Compostela, 27002 Lugo, Spain; eva.alonso.lopez@sergas.es (E.A.); rebeca.alvarino@usc.es (R.A.); amparo.alfonso@usc.es (A.A.); luis.botana@usc.es (L.M.B.); 3 Cell Culture Laboratory, Postgraduate Programme in Biotechnology, University of Vale do Taquari-Univates, Lajeado RS 95914-014, Brazil; diomarmitt@univates.br (D.M.); marcia.goettert@univates.br (M.I.G.); 4 BioISI-Biosystems and Integrative Sciences Institute, Faculty of Science, University of Lisbon, 1749-016 Lisbon, Portugal; hmgaspar@fc.ul.pt; 5 Center for Neuroscience and Cell Biology (CNC), University of Coimbra, 3004-517 Coimbra, Portugal; mcalpoim@gmail.com; 6 MARE—Marine and Environmental Sciences Centre, ESTM, Politécnico de Leiria, 2520-614 Peniche, Portugal

**Keywords:** breast cancer, red algae, oxidative stress, marine natural products, apoptosis, DNA damage, biological activities, MCF-7 cells

## Abstract

Marine natural products have exhibited uncommon chemical structures with relevant antitumor properties highlighting their potential to inspire the development of new anticancer agents. The goal of this work was to study the antitumor activities of the brominated diterpene sphaerodactylomelol, a rare example of the dactylomelane family. Cytotoxicity (10–100 µM; 24 h) was evaluated on tumor cells (A549, CACO-2, HCT-15, MCF-7, NCI-H226, PC-3, SH-SY5Y, SK-ML-28) and the effects estimated by MTT assay. Hydrogen peroxide (H_2_O_2_) levels and apoptosis biomarkers (membrane translocation of phosphatidylserine, depolarization of mitochondrial membrane potential, Caspase-9 activity, and DNA condensation and/or fragmentation) were studied in the breast adenocarcinoma cellular model (MCF-7) and its genotoxicity on mouse fibroblasts (L929). Sphaerodactylomelol displayed an IC_50_ range between 33.04 and 89.41 µM without selective activity for a specific tumor tissue. The cells’ viability decrease was accompanied by an increase on H_2_O_2_ production, a depolarization of mitochondrial membrane potential and an increase of Caspase-9 activity and DNA fragmentation. However, the DNA damage studies in L929 non-malignant cell line suggested that this compound is not genotoxic for normal fibroblasts. Overall, the results suggest that the cytotoxicity of sphaerodactylomelol seems to be mediated by an increase of H_2_O_2_ levels and downstream apoptosis.

## 1. Introduction

Due to demographic, societal, economic, and lifestyle changes, it is expected that the number of new cancer cases diagnosed each year will increase in the next decades [1] calling for the urgent development of new strategies to fight this burden. Several therapies have been approved for cancer treatments, including the use of apoptosis inducers. Apoptosis plays a crucial role in the development and maintenance of normal tissues homeostasis, tightly regulating programmed cell death by several biochemical and genetic pathways [2,3]. However, in several cancers, the expression of apoptosis inhibitors paved the way for cancer cells survival [2]. To counteract this problem, one possible approach can be the use of selective drugs to modulate the components of apoptotic pathways. However, this strategy has significant challenges since many of these targets involve complex protein–protein interactions. As a result, new strategies involving the generation of reactive oxygen species (ROS) are being implemented to overcome this challenge [4,5,6]. Reactive oxygen species play a dual role in cancer. They are involved in the mediation of protumorigenic signaling pathways linked to cell proliferation, survival, and adaptation to hypoxia. Conversely, these species can induce antitumorigenic signaling pathways, and, consequently, induce cell death by triggering oxidative stress [7]. This feature is behind the mechanism of action of several anticancer drugs mediate that by boosting ROS generation, increase of oxidative stress in tumor tissues promoting tumorigenic cells death by necrosis or apoptosis [8,9]. Various mechanisms have been proposed for ROS-induced apoptosis [10]. For instance, several studies have demonstrated that the excessive production of ROS mediates the mitochondrial membrane permeability transition pore complex, stimulating caspases, upregulating the death receptor 5, as well as signaling cascades linked to mitogen-activated protein kinase MAPK and extracellular signal-regulated kinase ERK pathways triggering apoptosis [10,11,12,13,14]. Among ROS, hydrogen peroxide (H_2_O_2_) has demonstrated to be an important mediator of apoptotic cell death [15,16,17]. Marine natural products (MNP), including terpenes, have exhibited potent and selective activities on different pharmacological targets by triggering intracellular signaling pathways linked to ROS production, such as H_2_O_2_, and apoptosis by mitochondrial mediated caspase dependent pathways [18,19,20,21].

**Figure 1:**
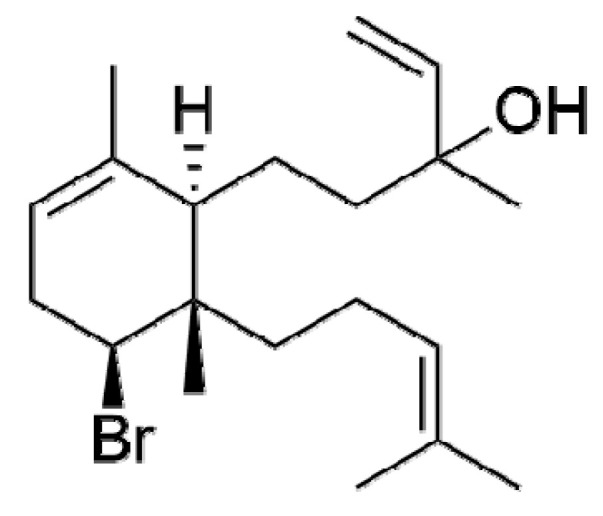
Chemical structure of sphaerodactylomelol.

The red alga *Sphaerococcus coronopifolius* Stackhouse, 1797, found along Atlantic and Mediterranean coasts, have revealed to be a great source of brominated cyclic diterpenes, some of them with antimicrobial, antifouling, antimalarial, and antitumor activities [22]. Sphaerodactylomelol (Figure 1) was identified for the first time in 2015, revealing antimicrobial and cytotoxic properties [23]. This bromoditerpene does not belong to the sphaerane family, commonly found in this species, but is an uncommon example of the dactylomelane family [24]. To date, there are only nine compounds of this class of diterpenes described in the literature and the knowledge on their biological activities is almost nonexistent [25]. Therefore, the goal of this work was to study the antitumor potential of sphaerodactylomelol on several in vitro human cancer cells, to define its selectivity and antineoplastic capacity. This is the first study that characterizes the intracellular signaling pathways linked to sphaerodactylomelol cytotoxicity activities, namely ROS production and apoptosis hallmarks.

## 2. Results

### 2.1. Cytotoxicity of Sphaerodactylomelol

Sphaerodactylomelol cytotoxicity was evaluated on several malignant cell lines and fibroblasts. The results are presented in Table 1.

Sphaerodactylomelol exhibited an IC_50_ range between 29.14 to 89.41 µM. The highest cytotoxicity was observed on 3T3 cells and the lowest on CACO-2 cells. Moreover, the cytotoxic effects of sphaerodactylomelol observed on colorectal cancer cells seemed to be more pronounced than the effects of standard antineoplasic drug 5-FU. Since it was not possible to observe a cytotoxic selectivity on the tested cellular models, MCF-7 cells were chosen for further studies to understand the mechanisms underlying the observed effects.

### 2.2. Production of H_2_O_2_

**Figure 2:**
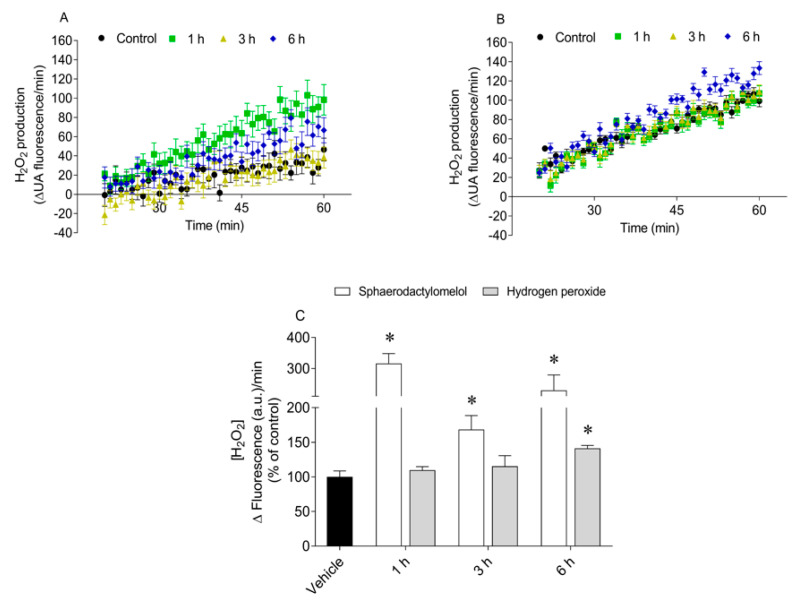
Production of H_2_O_2_ by MCF-7 cells treated with (**A**) sphaerodactylomelol (48 µM) and (**B**) H_2_O_2_ (200 µM) for 1, 3, and 6 h. MCF-7 H_2_O_2_ levels following 1, 3, and 6 h treatment with sphaerodactylomelol and H_2_O_2_ (**C**). Values represent mean ± SEM of at least three independent experiments carried out in triplicate. Symbols represent significant differences (*p* < 0.05) when compared to: * control.

The H_2_O_2_ levels produced by MCF-7 cells were quantified following the treatment with sphaerodactylomelol for 1, 3, and 6 h. The results are presented in Figure 2.

The treatment of MCF-7 cells with sphaerodactylomelol led to a significant increase of H_2_O_2_ levels as compared to control (Figure 2). The highest H_2_O_2_ production was observed following 1 h treatment triplicating the H_2_O_2_ levels relative to the control (316.3 ± 31.8% of control). As to H_2_O_2_ treatment, MCF-7 cells exhibited a significant increase on H_2_O_2_ levels following 6 h exposure.

### 2.3. Mitochondrial Membrane Potential

**Figure 3:**
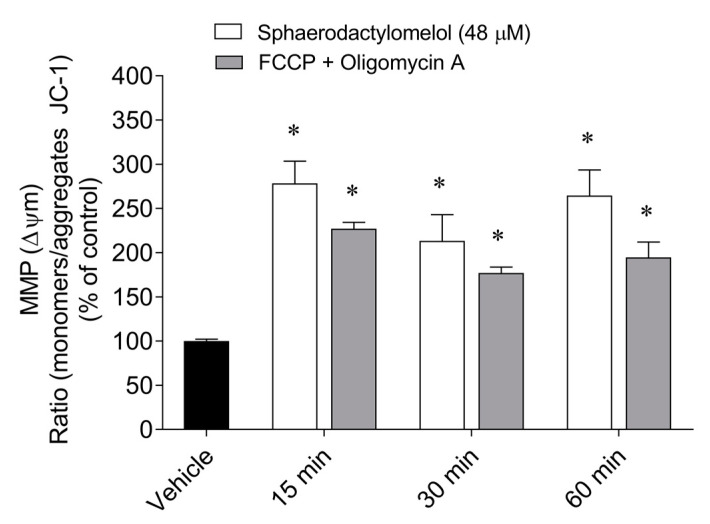
Mitochondrial membrane potential (MMP) of MCF-7 cells after treatment with sphaerodactylomelol (48 µM) and FCCP (2.5 µM) plus oligomycin A (1 µg/mL) for 15, 30, and 60 min. The results were expressed as the ratio between the monomers/aggregates of JC-1. Values represent the mean ± SEM of three or four independent experiments. Symbols represent significant differences (*p* < 0.05) when compared to: * control.

In order to understand if sphaerodactylomelol induced mitochondrial membrane potential (MMP) changes, MCF-7 cells were treated with the diterpene for 15, 30, and 60 min. The results are presented in Figure 3.

The treatment of MCF-7 cells with sphaerodactylomelol induced a marked depolarization of MMP after 15, 30, and 60 min, somehow similar to what was observed for FCCP and oligomycin (Figure 3).

### 2.4. Evaluation of Apoptosis Biomarkers 

**Figure 4:**
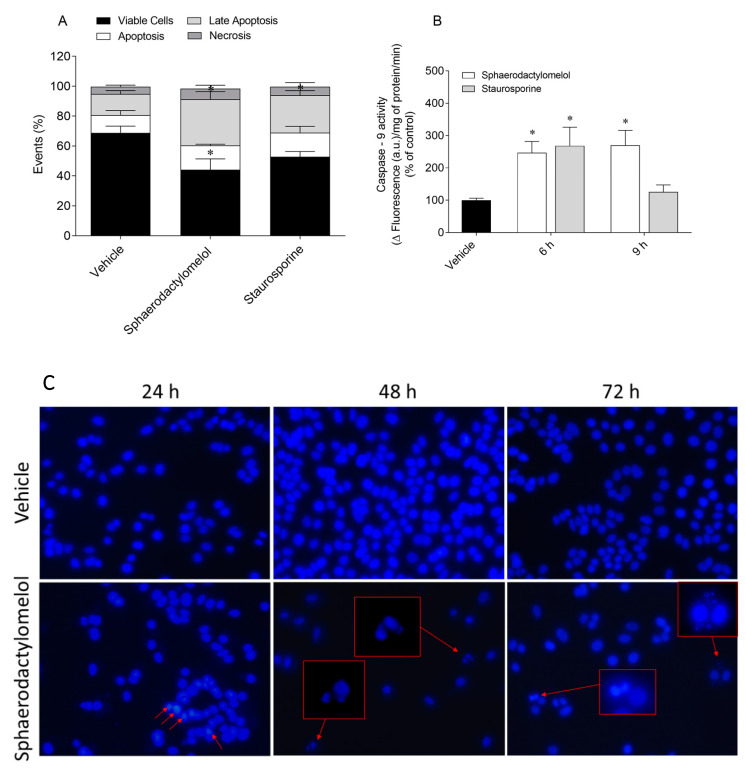
Sphaerodactylomelol effects on MCF-7 cells apoptosis biomarkers (**A**) externalization of phosphatidylserine, (**B**) Caspase-9 activity, and (**C**) DNA condensation and fragmentation. Values represent the mean ± SEM of three or four independent experiments. Symbols represent significant differences (*p* < 0.05) when compared to: * control. Images of 4′,6-diamidino-2-phenylindole (DAPI) stained cells were obtained using inverted fluorescence microscope at ×400. Arrows indicate alterations in DNA as comparing with control. The images are representative of one well of each situation tested.

Several biomarkers linked to cell death by apoptosis were studied, namely externalization of phosphatidylserine, Caspase-9 activity, and DNA condensation and/or fragmentation. The results are presented in Figure 4.

The MCF-7 cells exposition to sphaerodactylomelol for 24 h led to a significant decrease in the percentage of viable cells and a significant increase in the percentage of cells in late apoptosis stage when compared to the control (Figure 4A). Staurosporine, as positive control, also mediated a significant increase of cells population in late apoptosis stage. On the other hand, the percentage of cells in apoptosis and necrosis stages was similar to the control situation. Regarding Caspase-9 activity, the treatment with sphaerodactylomelol for 6 h and 9 h induced a marked effect in the Caspase-9 activity of MCF-7 cells (Figure 4B). As to staurosporine, the results clearly revealed that Caspase-9 activity is highest following 6 h exposure. One of the last events of apoptosis death is characterized by the DNA condensation and/or fragmentation. As illustrated in Figure 4C, sphaerodactylomelol treatment for 24, 48, and 72 h induced DNA condensation and fragmentation along the exposure time.

### 2.5. Genotoxicity Effects of Sphaerodactylomelol on L929 Fibroblasts

**Figure 5:**
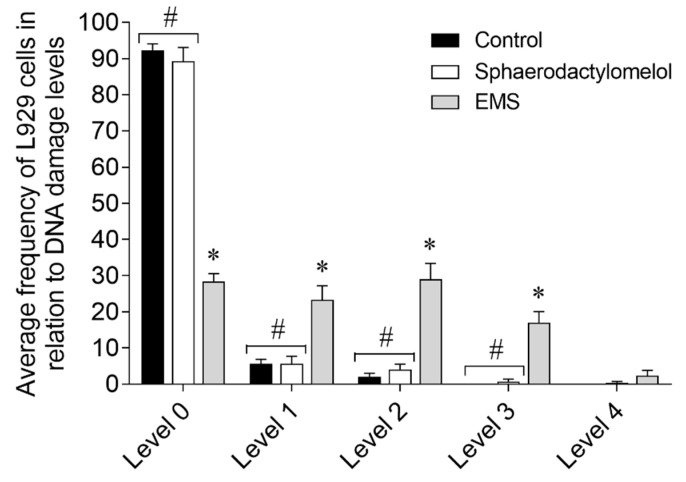
Frequency of L929 fibroblasts DNA change following exposure to sphaerodactylomelol (48 µM) and ethyl methanesulfonate (EMS) for 3 h. Values represent the mean ± SEM of three independent experiments. Damage index: Σ (comet class: 1, 2, 3, 4). 0, nucleus without DNA damage. Symbols represent significant differences (*p* < 0.05) when compared to: * control; # EMS.

To ensure compounds safety for therapeutic purposes, it is essential to determine their genotoxic effects [26]. Thus, the ability of sphaerodactylomelol to induce DNA damage was evaluated on L929 mouse fibroblasts (Figure 5).

The exposition of L929 fibroblasts to the bromoditerpene sphaerodactylomelol did not promote significant DNA damage when compared to control situation (Figure 5).

## 3. Discussion

Nature has played a fundamental role as a source of new anticancer therapeutic agents. In opposition to terrestrial sources, in the last decades, marine natural products have gained a growing interest, due to their distinct chemical features and new mechanisms of action [22]. The bromoditerpene sphaerodactylomelol does not belong to the usual sphaerane family commonly found in this species, but it is a rare example of the dactylomelane family, most of them characterized by a bridged oxide between C-7 and C-10, thus, leading to a 7-oxabicyclo[2.2.1]heptanes [24]. As the knowledge on the anticancer potential of these compounds is scarce, it was relevant to investigate sphaerodactylomelol cytotoxic effects. The results presented in this study revealed that sphaerodactylomelol induce cytotoxicity without selectivity on the different assayed malignant epithelial cells and normal cells. In fact, one of the main problems associated with cancer treatments is the unspecificity of the cytotoxic agent, which act on malignant cells and normal cells, resulting in serious side effects, such as acute and chronic toxicity. However, chemotherapy continues to be a corner stone in cancer therapeutics because it promotes a clear effect on cancer cells eradication [27,28]. Despite that sphaerodactylomelol did not exhibit selectivity for cancer cells, its therapeutic potential on cancer diseases should not be automatically discarded, because different approaches can be outlined in order to ensure its safe use, such as the development of drug delivery systems (e.g., nanoparticles) to direct its effects, reducing toxicity towards non-target cells, as well as the possibility to be used in combination with other anticancer drugs to potentiate the therapeutic effects [27,29]. As a result, it is important to understand the mechanisms that can be involved in the cytotoxic effects, and, thus, a thorough study was carried out using MCF-7 cells, a breast cancer cell line. The date attained revealed that exposure of MCF-7 cells to sphaerodactylomelol induced an increase in H_2_O_2_ levels, changes in mitochondrial membrane potential, and increased apoptosis levels, inducting Caspase-9 activation and DNA condensation and/or fragmentation. Altogether, these findings suggest that H_2_O_2_ mediated oxidative stress may be underling sphaerodactylomelol cytotoxicity. Several studies have demonstrated that compounds belonging to terpenes chemical class exhibit antiproliferative activities, which are generally associated to oxidative stress and apoptosis [18,19,20,21]. The controlled production of H_2_O_2_ on biological systems plays a central role in vital cellular processes [30,31]. However, the increase of its levels may promote mitochondrial dysfunction leading to cellular apoptosis [32,33,34]. H_2_O_2_ has the ability to downregulate and upregulate the expression of antiapoptotic (e.g., Bcl-2) and proapoptotic proteins (e.g., BAX), respectively, cytochrome C release, MMP loss, and increase of Caspase-3/-7/-9 activity [32,35,36,37]. These facts can support the hypothesis that the cytotoxic activities induced by sphaerodactylomelol are mediated by high levels of H_2_O_2_, which led to a clear depolarization of MMP, and a consequent stimulation of Caspase-9. Since Caspase-9 is involved in the formation of the multiprotein complex, apoptosome, it is expectable that the cytotoxic effects of sphaerodactylomelol may be mediated by intrinsic apoptotic pathways. Similar results were observed with other marine compounds, such as heteronemin, tuberatolide B, DDSD, and peroxy sesquiterpenoids, which promoted ROS generation and triggered apoptosis [18,19,21,38]. Spatane, a diterpenoid, isolated from the brown alga *Stoechospermum marginatum*, induced morphological changes, nuclear condensation, and fragmentation on B16F10 melanoma cells, also stimulating ROS production. The increase of these species led to a change in Bax/Bcl-2 ratio and triggered MMP loss, cytochrome c release, and activated the caspase-mediated apoptotic pathway [38]. Accordingly, Miyazato and co-workers (2016) verified that peroxy sesquiterpenoids derived from the soft coral *Sinularia* sp. induced cell death of HCT116 cancer cells by apoptosis-induction via caspase activation. Despite the results here described, further studies should be considered to understand the involvement of oxidative stress and apoptotic events in the cytotoxic effects mediated by sphaerodactylomelol. In order to verify if the increase of H_2_O_2_ levels play a critical role in the cytotoxic activities mediated by sphaerodactylomelol, it will be important to perform studies with known antioxidant compounds, such as N-acetyl-cysteine (NAC), which have the ability to neutralize these species. For instance, Koul and co-workers (2017) [39] proved that ROS generation mediated by cladosporol A on MCF-7 cells has a crucial role in cell death by the apoptotic mitochondrial pathway since, in the presence of NAC, the compound was unable to elevate ROS levels, failing to induce cell death. In addition, to increase the robustness of the data, the use of anticancer drugs (e.g., adriamycin, pacltitaxel) that induce cell death by increasing H_2_O_2_ levels, could also be used as controls. Furthermore, the study of other biomarkers associated with apoptosis (e.g., Caspase-8, cytochrome C release, Caspase-7, Bcl-2 family proteins levels) should also be evaluated to understand which pathways are activated, if death receptor (extrinsic) or mitochondrial (intrinsic), or both.

Summarizing, this was the first study regarding the characterization of the bromoditerpene sphaerodactylomelol cytotoxic potential. The compound induced cytotoxic effects in all the tested cellular models, which seems to be mediated by an increase of H_2_O_2_ levels, which in turn promoted changes in the mitochondrial membrane potential, Caspase-9 activation, and DNA condensation and fragmentation, triggering cell death by apoptosis.

## 4. Materials and Methods

### 4.1. Isolation of Sphaerodactylomelol Bromoditerpene

*Sphaerococcus coronopifolius* Stackhouse, 1797 was collected in Berlengas Nature Reserve (39°24′44.8″N 9°30′29.5″W), Peniche (Portugal), by scuba diving and immediately transported to MARE-Polytechnic of Leiria facilities. Samples were then cleaned to remove detritus, sands, and epibionts and freeze-dried. Sphaerodactylomelol was isolated as previously reported [23]. The purification was performed by chromatographic techniques, and structure elucidation by NMR and MS techniques. The compound was dissolved in DMSO (concentration below 0.2%) for biological assays accomplishment. The control situation was always treated with the highest concentration of DMSO as vehicle. Throughout the experiments controls and blanks were performed with cells resuspended in cells’ medium in presence and absence of DMSO, respectively.

### 4.2. Maintenance of Cell Cultures

Cell lines were acquired from ATCC and DSMZ and cultured according to the biobanks instructions. 3T3 (Fibroblasts; DSMZ: ACC173), A549 (Lung carcinoma; ATCC: CCL-185), and SH-SY5Y (Neuroblastoma; ATCC: CRL-2266) cells were cultured in DMEM/F-12 medium (Merck, Darmstad, Germany) supplemented with 10% serum bovine fetal (FBS) (Biowest, Riverside, MO, USA), GlutaMAX™ (Gibco, Gaithersburg, MD, USA), 100 UI/mL penicillin, and 100 μg/mL streptomycin (Biowest, Nuaillé, France). CACO-2 (Colorectal adenocarcinoma; DSMZ: ACC 169), HCT-15 (Colorectal adenocarcinoma; DSMZ: ACC 357), L929 (Fibroblasts; DSMZ: ACC 2), MCF-7 (Breast adenocarcinoma; DSMZ: ACC 115), NCI-H226 (Lung squamous cell carcinoma; ATCC: CRL-5826), and PC-3 (Prostate adenocarcinoma; ATCC: CRL-1435) were maintained in RPMI medium supplemented with 10% FBS, 100 UI/mL penicillin, and 100 μg/mL streptomycin. SK-MEL-28 cells (Melanoma; ATCC: HTB-72) were grown in EMEM medium (Sigma, St. Louis, MO, USA) supplemented with 10% FBS, 100 UI/mL penicillin, and 100 μg/mL streptomycin. Cells were dissociated with trypsin-ethylenediaminetetraacetic acid (Sigma, St. Louis, MO, USA), which was neutralized with the respective medium. To remove trypsin residues, cells were centrifuged at 290× *g* for 5 min at room temperature. Cells were then resuspended in new medium, split 1 to 10 and cultured in 25 cm^2^ T-Flasks. Cells were maintained in 5% CO_2_ and a humidified atmosphere at 37 °C.

### 4.3. Cytotoxic Activities on Malignant Cell Lines and on Normal Fibroblasts

The cytotoxicity of sphaerodactylomelol (0.1–100 µM; 24 h) was evaluated on cell lines (A549: 5 × 10^4^ cells/well; CACO-2: 5 × 10^4^ cells/well; HCT-15: 5 × 10^4^ cells/well; MCF-7: 5 × 10^4^ cells/well; NCI-H226: 1.5 × 10^4^ cells/well; PC-3: 2.5 × 10^4^ cells/well; SH-SY5Y: 5 × 10^4^ cells/well; SK-ML-28: 5 × 10^4^ cells/well; 3T3: 1.5 × 10^4^ cells/well) seeded in 96-well plates and incubated overnight. Untreated cells with DMSO were used as control and saponin (Sigma, Darmstadt, Germany), a cellular death inducer, as positive control. Cisplatin, tamoxifen, and 5-fluorouracil drugs (Sigma, Shanghai, China) were used as standards (0.1–500 µM; 24 h). The effects were estimated by the MTT assay with slight modifications [40] and the results expressed as IC_50_.

### 4.4. Hydrogen Peroxide (H_2_O_2_) Production

The production of H_2_O_2_ was measured using Amplex™ Red hydrogen peroxide Assay Kit (Molecular probes, Eugene, OR, USA). MCF-7 cells (5 × 10^4^ cells/well) were treated in 96-well plates with sphaerodactylomelol at IC_50_ concentration for 1, 3, and 6 h. H_2_O_2_ (200 µM) was used as positive control. The levels of H_2_O_2_ were calculated by the slope of the linear phase of fluorescence curve, and the results expressed in percentage of control (untreated cells with DMSO).

### 4.5. Mitochondrial Membrane Potential (MMP)

MMP was measured using JC-1 fluorescent probe (Molecular Probes, Eugene, OR, USA) according to the protocol developed by Silva and co-workers [41]. MCF-7 cells (5 × 10^4^ cells/well) were treated with sphaerodactylomelol at IC_50_ for 15, 30, and 60 min. Untreated cells were used as control. A conjugate solution with FCCP (2.5 µM) (Sigma, Rehovot, Israel) plus oligomycin A (1 µg/mL) (Sigma, St. Loius, MO, USA) was used as positive control. The formation of JC-1 aggregates (λ excitation: 490 nm; λ emission: 590 nm) and JC-1 monomers (λ excitation: 490 nm; λ emission: 530 nm) was measured in the plate reader for 30 min (Bio-Tek Synergy plate reader, Bedfordshire, UK). The results were expressed as the ratio of the monomers/aggregates of JC-1 in percentage of control (untreated cells with DMSO).

### 4.6. Apoptosis Biomarkers

#### 4.6.1. Annexin V and Propidium Iodide Staining

The translocation of membrane phosphatidylserine from the inner side of the plasma membrane to the surface (Annexin V) and the membrane integrity (propidium iodide) was evaluated by the means of Apoptosis Detection Kit (Immunostep, Salamanca, Spain) according to the manufacturer’s instructions. MCF-7 cells were seeded in 6-well plates (1 × 10^6^ cells/well) and incubated overnight. Cells were then treated with sphaerodactylomelol (IC_50_) for 24 h and stained with probes before analysis by flow cytometry. Untreated cells with DMSO were used as control. The antibiotic staurosporine (1 µg/mL) (Sigma, Rehovot, Israel) was used as positive control. Ten thousand events were attained with AMNIS imaging flow cytometer using the AMNIS INSPIRE™ software (Amnis Corporation v6.0, Luminex Corp, Austin, TX, USA). Data analysis was performed with the AMNIS IDEAS™ software. The results were expressed as percentage of events defined as viable, apoptosis, late apoptosis, and necrosis.

#### 4.6.2. Caspase-9 Activity

Caspase-9 activity was determined by the Caspase 9 Fluorimetric Assay Kit (Biovision, Milpitas, CA, USA) according to the manufacturer’s instructions. MCF-7 cells were cultured in 6-well plates (1 × 10^6^ cells/well) and treated with sphaerodactylomelol (IC_50_) concentration for 3, 6, and 9 h. Untreated cells were used as control and staurosporine (1 µg/mL) was used as an apoptosis inducer. The fluorescence was measured (λ excitation: 400 nm; λ emission: 505 nm) during 90 min at room temperature. Caspase-9 activity was determined by the slope of the fluorescence resulting from 7-amino-4-(trifluoromethyl) coumarin accumulation (∆fluorescence (u.a)/mg of protein/min) and expressed in percentage of control (untreated cells with DMSO).

#### 4.6.3. Nuclear Condensation and/or DNA Fragmentation

Nuclear condensation and/or DNA fragmentation were determined by DAPI staining as previously described [41]. MCF-7 cells were seeded in 6-well plates (1 × 10^6^ cells/well) and incubated overnight. Cells were then exposed to sphaerodactylomelol (IC_50_) during 24, 48, and 72 h. Ending this time, cells were staining with DAPI and observed in a fluorescence inverted microscope (ZEISS Axio, VERT. A1, equipped with an AxioCam MRC-ZEISS camera, Oberkochen, Germany). Untreated cells with DMSO were used as control. The images presented are representative of each individual experiment.

### 4.7. L929 Fibroblast Genotoxicity Studies

The genotoxic effects of sphaerodactylomelol was studied on L929 mouse fibroblasts according to the method developed by Singh and co-workers [42] with slight modifications. Cells were cultivated on T-flasks, and posteriorly in 12-well plates (2 × 10^4^ cells/well) for 12 h at 37 °C. L929 cells were then treated with sphaerodactylomelol at a concentration of 0 (control) and at 50 µM for 3 h. Ethyl methanesulfonate (200 µg/mL) (Sigma, St. Loius, MO, USA) was used as positive control. Briefly, 15 µL of the cells’ suspension was suspended in 90 µL of low-melting-point agarose (37 °C). The suspension was then spread on a previously prepared thin layer of UltraPureTM agarose slide, covered with coverslip, and maintained at 4 °C for 10 min. Thereafter, the slides were immersed into a lysis solution (2.5 M NaCl, 100 mM Na2EDTA, 10 mM Tris, 1% Triton×100, and 10% DMSO; pH 10.0) at 4 °C for 24 h. The slides were then subjected to electrophoresis and stained with silver nitrate. Results were analyzed by optical microscopy (400X). The DNA damage induced on L929 cells was quantified as the amount of DNA released from the nucleus. Analysis of 100 cells randomly chosen and non-overlapping was performed. Cells were visually scored and classified into five levels, according to tail size formed by DNA fragments.

### 4.8. Data and Statistical Analysis

Results are presented as mean ± standard error of the mean (SEM) or standard deviation (SD) and IC_50_ determined by the analysis of non-linear regression by means of the equation: y = 100/(1 + 10(X − LogIC_50_)). At least three independent experiments in triplicate were carried out. One-way analysis of variance (ANOVA) with Dunnett’s multiple comparison of group means to determine significant differences relatively to control treatment was accomplished. For the remaining multiple comparisons, the Tukey’s test was applied. When applicable, the Student’s *t*-test was used to observe the differences between the means of control and treatments. Differences were considered significant at level of 0.05 (*p* < 0.05). The analyses were accomplished using IBM SPSS Statistics 24 (IBM Corporation, Armonk, NY, USA) and GraphPad v5.1 (GraphPad Software, La Jolla, CA, USA) software.

## Figures and Tables

**Table 1:** Values of IC_50_ (μM) determined for sphaerodactylomelol (0.1–100 μM) and standard drugs (0.1–500 μM) on tumorigenic cell lines and fibroblasts following 24 h treatment. The values represent the mean plus standard deviation. At least three independent experiments in triplicate were carried out.

	A549	CACO-2	HCT-15	MCF-7	NCI-H226	PC-3	SH-SY5Y	SK-MEL-28	3T3
Sphaerodactylomelol	71.99 ± 15.65	89.41 ± 31.67	46.25 ± 10.05	47.19 ± 23.97	39.54 ± 9.22	58.21 ± 13.98	33.04 ± 8.76	40.51 ± 11.30	29.14 ± 3.64
Cisplatin	271.1 ± 111.10	-	-	-	172.9 ± 84.42	267.2 ± 67.14	13.92 ± 6.70	51.52 ± 6.87	137.0 ± 3.64
Tamoxifen	-	-	-	27.19 ± 21.53	-	-	-	-	15.34 ± 1.55
5-Fluororacil	-	382.7 ± 32.95	155.5 ± 44.04	-	-	-	-	-	344.7 ± 19.69

## Data Availability

The data presented in this study are available on request from the corresponding author.

## Sample Availability

Samples of the compound are available from the authors.
